# Whole-Genome Sequence of Fish-Pathogenic Enterococcus faecalis Strain BFFF11

**DOI:** 10.1128/MRA.01447-19

**Published:** 2020-02-13

**Authors:** Tasmina Akter, M. Mahbubur Rahman, Alfred Chin Yen Tay, Rakib Ehsan, M. Tofazzal Islam

**Affiliations:** a Institute of Biotechnology and Genetic Engineering, Bangabandhu Sheikh Mujibur Rahman Agricultural University, Gazipur, Bangladesh; b Marshall Centre for Infectious Diseases Research and Training, School of Biomedical Sciences, University of Western Australia, Perth, WA, Australia; University of Maryland School of Medicine

## Abstract

A fish-pathogenic bacterium, Enterococcus faecalis strain BFFF11, was isolated from a tilapia suffering from streptococcosis in a fish farm in the Gazipur district of Bangladesh. The whole genome of this strain, BFFF11, was 3,067,042 bp, with a GC content of 37.4%.

## ANNOUNCEMENT

Enterococcus faecalis is an opportunistic pathogen that causes diseases in plants, animals, and humans (1). Recently, it was reported as a virulent pathogen of tilapia (2). This report describes the whole-genome sequence of fish-pathogenic E. faecalis strain BFFF11.

E. faecalis strain BFFF11 was isolated on KF streptococcal agar (HiMedia Laboratories Pvt. Ltd., India) from a diseased tilapia in Bangladesh (23.9999°N, 90.4203°E). The genomic DNA was extracted from an overnight nutrient broth culture (Liofilchem S.r.l., Italy) (2) by using the GeneJET genomic DNA purification kit (Thermo Fisher Scientific, USA), and the quantity was checked with a NanoDrop spectrophotometer (Thermo Fisher Scientific). For genomic sequencing, 1 ng of genomic DNA was fragmented using 5 μl of Tagment DNA enzyme with 10 μl of Tagment DNA buffer (Illumina, Inc., San Diego, CA, USA) at 55°C for 10 min, followed by 10 min of neutralization with 5 μl of Neutralize Tagment buffer and a 12-cycle PCR procedure for barcoding nucleotide sequence incorporation. The barcoded DNA library was purified using 30 μl of AMPure XP beads (Beckman Coulter, Inc., Australia). The concentration of the barcoded DNA library was normalized to 5 nM, and the library was denatured with 0.2 N NaOH and further diluted to 13 pM. A 600-cycle sequencing procedure was performed using a MiSeq sequencer (Illumina, Inc.).

The Bacterial Analysis Pipeline v.1.0.4 was used for initial identification of bacteria (3). The sequence adaptors were removed from the raw sequencing reads with Trimmomatic v.0.38 (4), and quality filtering was done using PRINSEQ v.0.20.3 (5). *De novo* assembly was performed using quality reads into draft genomes with SPAdes v.3.9.0 (6). The QUAST v.5.0.2 tool was used for quality evaluation of the assembled draft genome (7). Seventy-one contigs were used for annotation of the draft genome using Prokka v.1.11.0 (8). The lengths of the smallest and largest contigs were 211 and 660,287 bp, respectively. The annotated chromosome length, GC content, and *N*_50_ value of the assembled genome were 3,067,042 bp (64 contigs), 37.4%, and 343,888 bp, respectively. The open reading frames of the genome were predicted and annotated using Rapid Annotations using Subsystems Technology (classic RAST FIGfams v.70) (9), which showed 357 subsystems with 49% coverage of the total subsystems, 2,870 protein-coding sequences, and 66 RNA genes.

By using the ResFinder database (10), the macrolide resistance gene *lsa*(A) was found in the contig at positions 334167 to 335663, with 98.73% identity (using the following settings: threshold identity, 90%; minimum length, 60%). No plasmid replicon was identified in the genome by using the PlasmidFinder database (minimum values for threshold identity and coverage were 95% and 60%, respectively) (11). The whole-genome sequence of fish-pathogenic E. faecalis strain BFFF11 may provide additional information for the diagnosis and prevention of streptococcosis in fish.

### Data availability.

The complete whole-genome sequence of E. faecalis strain BFFF11 has been deposited in GenBank under accession no. CP045918, and the raw data are available under accession no. SRX7484814. The data are available under BioProject accession no. PRJNA587873 and BioSample accession no. SAMN13220412.
